# The Localization and Characterization of Ischemic Scars in relation to the Infarct Related Coronary Artery Assessed by Cardiac Magnetic Resonance and a Novel Automatic Postprocessing Method

**DOI:** 10.1155/2015/120874

**Published:** 2015-10-12

**Authors:** Leik Woie, Kjersti Engan, Trygve Eftestøl, Alf Inge Larsen, Stein Ørn

**Affiliations:** ^1^Department of Cardiology, Stavanger University Hospital, 4068 Stavanger, Norway; ^2^Department of Electrical and Computer Engineering, University of Stavanger, 4036 Stavanger, Norway; ^3^Department of Clinical Science, University of Bergen, 5020 Bergen, Norway

## Abstract

*Aims*. The correspondence between the localization and morphology of ischemic scars and the infarct related artery (IRA) by use of cardiac magnetic resonance imaging and a novel automatic postprocessing method. *Methods and Results*. Thirty-four patients with one-year-old single IRA myocardial infarction were examined. Endocardium, epicardium, and the point where right and left ventricles are coinciding were manually marked. All measurements were automatically assessed by the method. The following are results with manual assessments of scar properties in parenthesis: mean scar size (FWHM criterion): 7.8 ± 5.5 as %LV (17.4 ± 8.6%); mean endocardial extent of infarction: 44 ± 26° (124 ± 47°); mean endocardial extent of infarction as %LV circumference: 9.7 ± 7.0% (34.6 ± 13.0%); and mean transmurality: 52 ± 20% of LV wall thickness (77 ± 23%). Scars located in segments 1, 2, 7, 8, 13, and 14 by use of the automatic method were 96–100% specific for LAD occlusion. The highest specificities of RCA and LCX occlusions were segment 4 with 93% and segment 6 with 64%, respectively. The scar localization assessed automatically or manually was without major differences. *Conclusion*. The automatic method is applicable and able to assess localization, size, transmurality, and endocardial extent of ischemic scars.

## 1. Background

The 17-segment model of the American Heart Association (AHA) is important for assessing regional function and pathology in many imaging modalities of the heart [1]. The assignment of individual myocardial segments to coronary artery territories as proposed by the AHA is based on studies in 35 patients by use of radiography with technetium-99m sestamibi and balloon occlusion of a coronary artery [2].

The assignment of myocardial scar to coronary artery territories has also been investigated in other studies during percutaneous coronary occlusion and use of technetium-99m sestamibi [3, 4]. There is concern that the low spatial resolution of technetium-99m perfusion imaging used to validate the original AHA annotation may contribute to the observed discordance between coronary perfusion and myocardial segments. Late gadolinium-enhancement cardiac magnetic resonance (LGE-CMR) has higher spatial resolution than nuclear perfusion imaging [5] and has also been used to investigate the correspondence between the coronary arteries and their myocardial perfusion areas according to the 17-segment model [6, 7]. In these studies, significant discordance between the AHA proposal and the observed myocardial scars and coronary distribution was found in segments 12, 15, and 16. In addition to manual marking of endo- and epicardium, analysis of LGE-CMR images by current methods also requires manual tracing of the scars, their location, and transmurality. We have estimated the additional work for these tasks to last about 20 minutes. According to our experience, these procedures are also relatively operator-dependent and difficult to practice on a busy daily basis.

The aim of this study is to demonstrate the clinical applicability of a new automatic method by assessment of the correspondence between the infarct related artery (IRA) and the segmental localization of the scar and compare the results with the AHA proposal and other studies that show the relationship between the IRA and the location of scars.

## 2. Methods

### 2.1. Study Population

We evaluated 41 consecutive patients with healed myocardial infarction (MI) caused by 1 vessel disease and acute ST-segment elevation myocardial infarction (STEMI) successfully revascularized with primary percutaneous coronary intervention (pPCI) one year prior to LGE-CMR examination [8]. After retrieval of the LGE-CMR images from the local PACS (Picture Archive and Communication System), 7 patients were excluded due to low resolution, giving a study population of 34 patients. Only patients with acute proximal, mid-, or distal occluded single-vessel disease were included. Patients with previous MI and evidence of reinfarction during the first year after PCI were excluded. All patients had stable sinus rhythm at the time of LGE-CMR examination. The Regional Ethics Committee approved the study and all patients provided written informed consent prior to inclusion.

### 2.2. LGE-CMR Protocol

The LGE-CMR protocol has been described previously [8]. CMR was performed with a 1.5 T Philips Intera R 8.3. To achieve maximum signal, a 2D sequence without parallel imaging (SENSE) was used. Automatic B0 volume shimming of the heart imaging volume was performed prior to the first sequence acquisition. The cardiac coil consisted of five anterior elements. Coil roll-off was considered insignificant within the radius of the elements until 12 cm from the chest wall. Functional assessments of left ventricular ejection fraction (LVEF) and volumes were performed according to current recommended standards with the use of steady-state, free precession sequence covering the whole left ventricle (LV) with 8 mm thick short-axis slices and interslice gaps of 2 mm. Assessment of LV volumes was performed on full short-axis datasets in a random, blinded fashion, using the View Forum Software (Philips Medical Systems, Best, The Netherlands). Indexes for LV volumes were obtained by correcting for body surface area.

After assessments of volumes and LVEF were performed, a gadolinium-based contrast agent, Omniscan, was administered intravenously at a dosage of 0.25 mmol/kg [11]. The sequence was a classical 2D sequence without any parallel imaging and typical 12 sec. breath hold. Delayed hyperenhanced images were acquired 10–15 minutes following Gd contrast infusion, using an inversion-recovery-prepared T1 weighted gradient-echo (T1-GRE) sequence, TR 4.1 ms (range 4.0–4.2 ms), and TE 1.3 ms. Images were acquired with a pixel size of 0.82 × 0.82 mm^2^, covering the whole ventricle with short-axis slices of 10 mm thickness, without interslice gaps (typically 12 slices). Inversion time was individually adapted aiming to null normal myocardium (typically 200–300 ms). Mean heart rate was 57.7 bpm (range 52–68).

### 2.3. Processing of LGE Images

**Figure 1:**
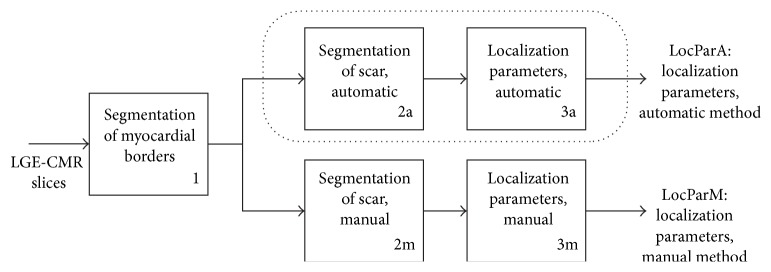
The dotted line including box 2a and box 3a illustrates the automatic method for finding localization parameters for the myocardial scar, and the output is named LocParA (Localization Parameters Automatic). Box 2a represents the 3DmaxSI method from [14], which corresponds to the Full-Width-Half-Maximum (FWHM) criterion. Box 3a corresponds to the automatic localization parameter algorithm, first described by the authors in [13]. 2m and 3m correspond to manual demarking of the scar followed by a manual interpretation of the localization of the scar according to the AHA standard of 17 segments and the transmurality. The result is named LocParM (Localization Parameters Manual). In this paper, the findings of LocParA and LocParM are both compared to knowledge about which artery caused the infarction in the different patients.

The images were retrieved in DICOM (Digital Imaging and Communications in Medicine) format with 512 × 512 pixels and a bit depth of 12, which represents DICOM format with up to 2^12^ = 4096 values of signal intensity (SI), but most of our patients had 256–1024 different SI values. The image analysis was done using in-house-developed software programs written in MATLAB. As a first step in assessing the localization parameters of the myocardial scar, the endocardial and epicardial borders must be found, illustrated with box 1, Figure 1. There are research groups working on the problem of doing automatic segmentation of the myocardium in LGE-CMR images [9, 12]. As of today there are no thoroughly tested, publically available automatic methods that are functioning well enough, but we are optimistic that this problem might very well be solved in the future. In this work, the endocardium and epicardium borders are manually annotated as a consensus of two cardiologists (Stein Ørn and Leik Woie). This could be replaced by a semiautomatic or fully automatic method when available. All LGE-CMR was performed one year after acute MI and no microvascular obstruction was present at this time.

**Figure 2:**
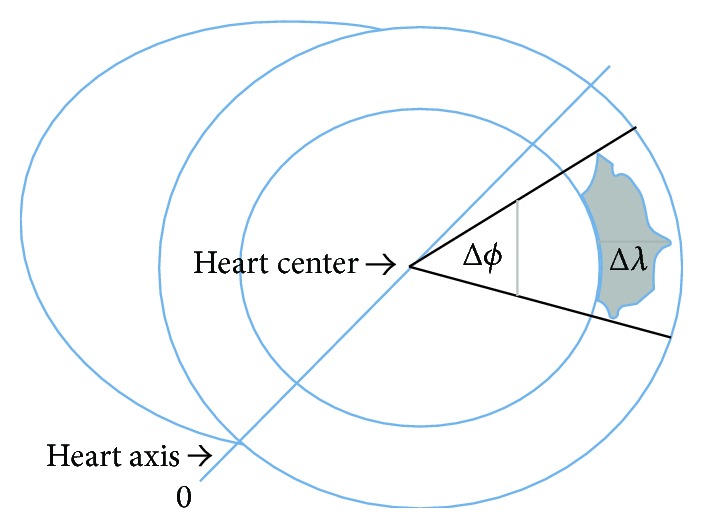
Diagrammatic drawing of scar measurements. Most patients have 6–9 slices, and the drawing is a transversal cut of right and left ventricle of one slice. The phase *ϕ*(*x*) of any pixel *x* is defined as the angle (ranging from 0 to 360°) between the line joining the pixel and the heart center and the heart axis. The endocardial extent of infarction (Δ*ϕ*) of a scar is drawn through the part of the scar with the shortest and longest phase as shown in the scar of the drawing. The radial position, *λ*(*x*), of any pixel *x* within the myocardium is a number between 0 and 1 representing the relative distance within the myocardial borders. 0 represents endocardium and 1 epicardium. Δ*λ* is defined as the distance between *λ* = 0 and maximum *λ*. Only scars adherent to endocardium with *λ* = 0 are used in the current study.

In accordance with the recommendation by AHA, only transverse slices containing myocardium in all 360° were selected for the assessment of the myocardium. This is due to the complex mixing of myocardium and connective tissue at the base of the heart, especially the septum [1]. The next step for the program was to compute the average position of the centroids (in MATLAB) of all pixels of the demarked myocardium, which was assessed for each slice. Thereafter the heart axis was found as the line between the centroid and the manually marked point where right and left ventricles are coinciding (Figure 2). If this point was not visible, heart axis was marked in reference to the previous slice where both ventricles were visible. The heart axis defined the *ϕ* = 0 axis and the angle was defined from 0 to 360° in a counterclockwise direction [13]. After visually confirming the existence of a scar in at least one of the slices, pixels from all slices are automatically merged. The maximum SI from the merged pixels is denoted by* 3DmaxSI* and this value is used to identify scar tissue in all slices [14]. Scar or infarct size is defined as pixels with SI ≥ 50% of* 3DmaxSI* and that is the Full-Width-Half-Max (FHWM) criterion. Thereafter, a scar segment is defined as a group of at least two connected pixels labelled as a scar, knowing that this small hyperenhancement could also be artefact, vessel, muscular cleft, or similar structures. For every pixel (*x*) between the endocardial and epicardial border, *ϕ*(*x*) is the angle between the heart axis and a radial line from the center of the myocardium through the pixel. The circumferential involvement of any scar is called the endocardial extent of infarction (Δ*ϕ*) and it is defined as the smallest angle (sector) containing all the pixels defined as scar segment (Figure 2). It is important to distinguish the endocardial extent of infarction (Δ*ϕ*) from the endocardial extent of infarction as %LV circumference, which is the sum of all Δ*ϕ* divided by 360*∗*number of slices with one scar [15, 16].

For every pixel (*x*) between the endocardial and epicardial border, we also define *λ*(*x*) to be a value between 0 and 1, indicating if the pixel is close to the endocardial border (close to 0) or close to the epicardial border (close to 1). In short, this is done by again using the radial line from the center of the myocardium through the pixel (*x*). The pixels inside the myocardium along this line (in practice, a small angular segment around this line) are given a value from 0 to 1 so that even if the thickness of the myocardium is not fixed, the *λ*(*x*) value will always give an interpretation of how close that specific pixel is to the endocardium (or epicardium) wall (Figure 2). The difference between the maximum *λ*(*x*) and minimum *λ*(*x*) is called the transmurality of the scar, Δ*λ*. For every patient, the sum of scar areas was divided by the sum of sectors surrounding the scars and presented as the scarring % [17].

All scars with minimum *λ* = 0 were set as the border of endocardium, and values with minimum *λ* > 0 are scars without connection to endocardium.

**Figure 3:**
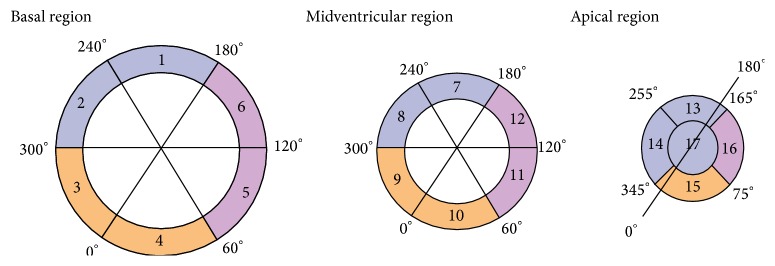
The figure is a diagrammatic representation of the short-axis views of the basal, midventricular, and apical regions of the ventricles in accordance with the AHA 17-segment model. The ventricles are divided into thirds along the long axis of the ventricles. The territories of left anterior descending artery (LAD), right coronary artery (RCA), and left circumflex artery (LCX) have their own color and angle.

The left ventricle is divided into equal thirds perpendicular to the long axis of the heart. This generates three circular sections of the left ventricle named basal, mid-, and apical regions (Figure 3). The basal and midregions are divided into 6 segments of 60° as recommended by the AHA-17 model [1]. Only four segments of 90° are used for the apical region, and the border between segments 14 and 15 is shifted by 15° in a clockwise direction compared to the heart axis. If there were 6 slices, 2 slices were assigned to the apical, mid-, and basal regions. If the patient had 7 slices, slice number 7 was assigned to midregion, and, with 8 slices, slice number 7 was assigned to midregion and slice 8 to basal region. The same order was applied if the patients had 10 or 11 slices. The scar areas were assigned to the 17-AHA model based on the localization *ϕ*(*x*). Note that a contiguous scar area can be split into several of the AHA model segments. Each individual pixel of the scar is assigned to the corresponding AHA model segment. The demarcation of the myocardial border and the marking of the point of the heart axis were done manually as previously described.

Segment 17 is the area of myocardium beyond the end of the left ventricular cavity and it is not included in our automatic analyses. However, the localization of scars in this segment was visually assessed by use of the 2-chamber long-axis views, and a scar was present if LGE was present in more than 50% (>180°) of the circumferential extent of that segment. Except for segment 17, all calculations involving allocation of scar pixels to the AHA segments and the size, transmurality, and endocardial extent of the infarction are done entirely automatically [13].

**Figure 4:**
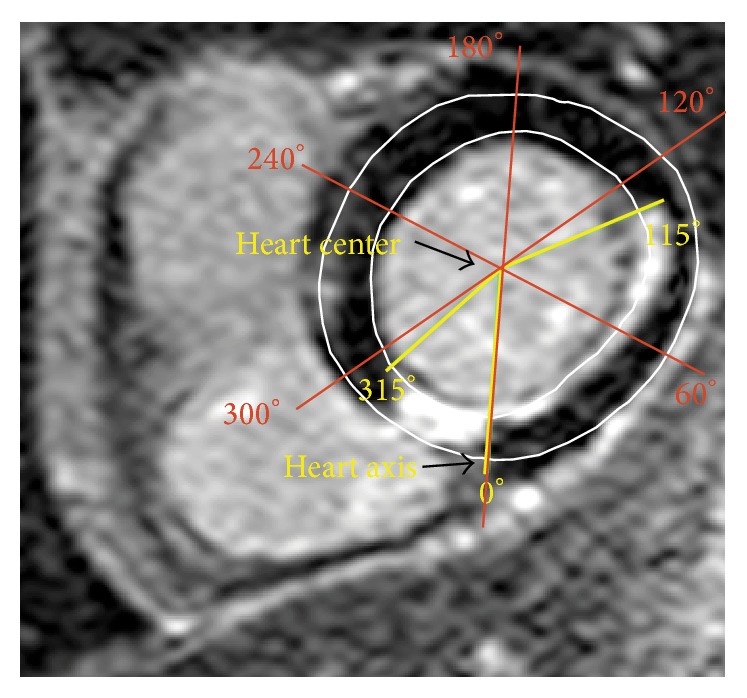
Example of one slice with manual marking. The slice has visual marking of the heart center and the heart axis as the line between the heart center and the point where right and left ventricles are coinciding. The slice is taken from the basal cavity and the scar is located between 315° and 115°. The location of the scar according to the 17-AHA is for segment 3 scars between 300° and 360°, for segment 4 between 0° and 60°, and for segment 5 between 60° and 120°. In this example, the part of segment 3 is 360° − 315° = 45°, segment 4 is 60°, and segment 5 is 115° − 60° = 55°.

In order to compare the results found by this new automatic method, a manual method was employed. One representative slice from the apical, mid-, and basal cavity of all patients was selected and scars were manually allocated to the coronary artery territories according to the 17-AHA proposal. First the heart center was visually marked (Figure 4). The start and end of the circumferential involvement of the scar were also manually marked, as shown by the example in Figure 4. The angle marked was assessed by use of OsiriX v.5.5.2 32-bit software. Localization of the scars according to the 17-AHA model was based on the same angles as used for the automatic method (Figure 3). Manual assessment of scar size and transmurality as the distance between endocardium and maximum transmurality of the scar and as percent of LV thickness has also been performed.

### 2.4. Statistics

Statistical analyses were performed using SPSS version 21. Continuous data are reported as means and standard deviations, and categorical data are reported as counts and proportions. Differences in the distribution of continuous variables between all three patient groups were tested by the Kruskal-Wallis test. For categorical data, the Chi-square test was used. All tests were two-tailed and a *p* value of ≤0.050 was considered significant.

## 3. Results

A total of 34 patients were included in this analysis (Table 1). All patients had normal kidney function (GFR: 97 ± 24 mL/min/1.73 m^2^).

Only scars with minimum *λ* = 0 were used for the assessment of scar localization in order to include only ischemic scars attached to the endocardium. A total of 257 (median = 8, minimum 6–maximum 9) slices were analysed in addition to the long-axis views of all patients. There were no significant differences of baseline characteristics in patients with LAD, RCA, or LCX as IRA, except the use of aldosterone antagonists, Table 1.

Cardiac magnetic resonance characteristics assessed by automatic and manual methods are shown in Table 2. There were no significant differences in CMR characteristics except manual assessment of mean Δ*ϕ* and endocardial extent of infarction as %LV circumference in patients with LAD and RCA as IRA.

**Figure 5:**
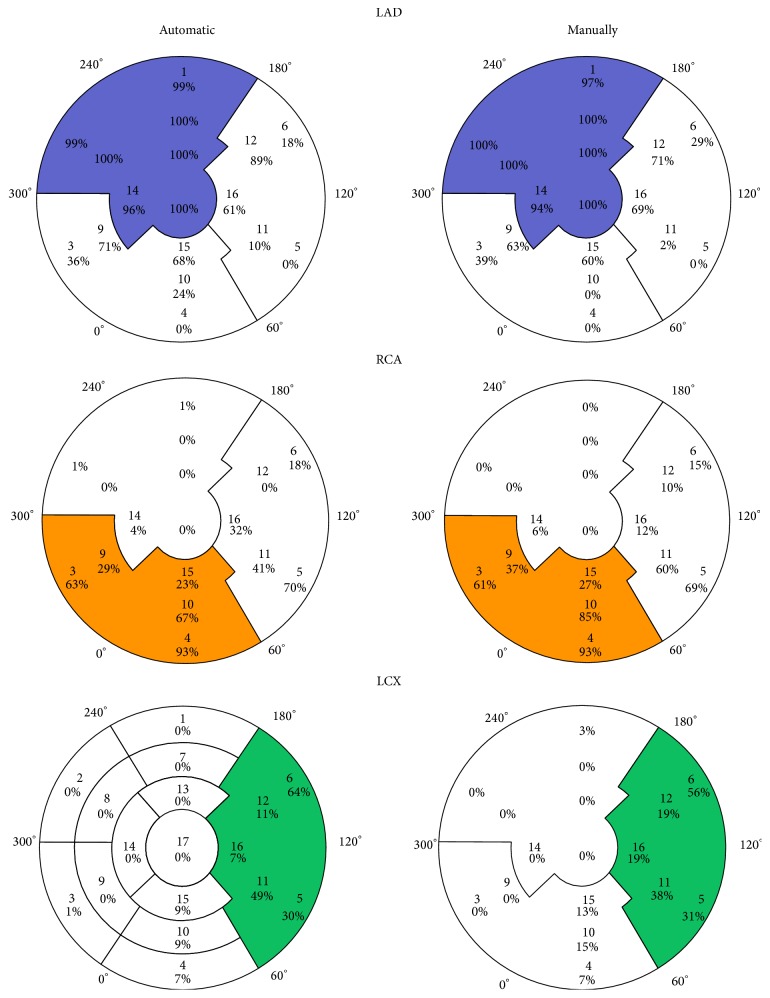
Distribution of LGE-CMR according to the AHA proposal and infarct related artery (IRA) by automatic and manual assessment. The automatic assessment is calculated from all transverse slices from the basal, mid-, and apical regions, whereas the manual assessment is calculated from one representative transverse slice from the basal, mid-, and apical cavities. For all scars, the endocardial extent of infarction (Δ*ϕ*) is assessed (0°–360°) and the total sum is calculated and allocated to each segment. The percent marking is the percentage of the total sum of Δ*ϕ* for each of the 16 segments used in this study; that is, sum of all percentages for one segment is equal to 100%. The manual assessment of segment 17 is also included. The percentage demonstrates the agreement of the observed LGE within each IRA segment as proposed by AHA. The manual assessment of segment 17 is also included in the automatic assessment.

Automatic assessments of scars according to the AHA segmentation of LGE-CMR images in relation to the IRA are presented in Figure 5 and Table 3. By visual assessment of segment 17, a total of 11 patients had scars with LAD as IRA. Therefore segment 17 has 100% agreement with the AHA proposal. The scars of patients with the same IRA have been selected and the locations of the scars are presented as the percentage of the total sum of circumferential involvement of every segment (Figure 5 and Table 3). The percentage demonstrates the agreement of the observed scar, within each IRA zone as proposed by the AHA, assessed by the automatic and manual methods.

## 4. Discussion

By LGE-CMR imaging of myocardial scars one year after successful revascularization of single-vessel occlusion by use of PCI, only 4 segments were 100% specific for LAD occlusion and none for RCA or LCX occlusion.

The scar or infarct size assessed with the automatic method used the FHWM criterion, and the size is much smaller than the size assessed with manual marking (Table 2). We used the FWHM criterion since it has been validated against histology in an animal model [18], and the FWHM technique is also the most reproducible method [19], but there is no consensus about which method should be used [10, 20]. The manually marked scars in the current study were 17.4 ± 8.6% as %LV, and this is comparable with manually marked scars in the study by Ortiz-Pérez et al. [15] where the infarct size as %LV mass was 22.4 ± 11.5. The automatic method is able to analyse each scar separately, while the visual assessment in most cases only locates one scar per slice. The automatic scar assessment uses the FWHM criterion, while manual scars are assessed visually. These differences in techniques and scar criteria explain why scar size, mean Δ*ϕ*, endocardial extent of infarction as %LV circumference, and mean Δ*λ* are bigger when the manual method is used.

It has been demonstrated that the degree of hyperenhancement caused by LGE-CMR portrays the damage caused by the IRA [15]. The 17-segment AHA model is a well-recognized recommendation for nomenclature and location of myocardial segments [1]. According to the maximum specificity of CMR hyperenhancement, the LAD territory assessed by our method is in accordance with the proposed AHA locations and has a specificity of 96–100% (Figure 5, Table 3). In addition, our data indicates that segments 9, 12, 15, and 16 might be considered as LAD territory due to specificity of 71, 89, 68, and 61%, respectively (Figure 5, Table 3). When we compare the location of the scars calculated on the basis of automatic or manual assessment, no major differences are seen. Segments 12, 15, and 16 as LAD territory have also been suggested by other studies [6, 7]. Despite these differences in techniques, our automatic method shows similar results, especially that all 3 studies showed segments 12, 15, and 16 to be discordant with the 17-AHA proposal. These findings suggest that our automatic method is applicable.

LAD or RCA supplies segment 9 [4]. In our study LAD has specificity of 71% and RCA 29% to be IRA of segment 9 (Figure 5, Table 3). A trend of a higher degree of collateral flow in patients with RCA occlusion has been shown [7]. This may cause a reduction in the size of the myocardium supplied by RCA. In addition to large individual variability of coronary artery distribution, the most likely cause of the discordance of segments 15 and 16 is the coronary blood supply of the apical segments 13–17 that is dominated by LAD [4, 6]. Also the assignment of segments 9 and 12 to the territory of LAD demonstrates the large amount of myocardium supplied by this artery. The consequences of large LAD territory are in accordance with the findings shown in Table 2. When the automatic method is used comparing LAD and RCA territory, LAD as IRA causes lower LVEF, bigger volumes, larger scars, larger mean endocardial extent of infarction measured as angle (Δ*ϕ*), and endocardial extent of infarction as %LV circumference, although the differences are not statistically significant in this small study. When the manual method is used, LAD as IRA causes significantly larger mean Δ*ϕ* and endocardial extent of infarction as %LV circumference compared with RCA as IRA. These findings are in accordance with the study by Ortiz-Pérez et al. [7], where the differences also were statistically significant. These findings also support the applicability of the automatic methodology.

Our data of RCA territory is in agreement with the proposed AHA locations for segments 3, 4, and 10 with a specificity of 63, 93, and 67%, respectively (Figure 5, Table 3). RCA or LCX supplies segment 5 [4]. In our study, RCA has a specificity of 70% and LCX 30% to be IRA of segment 5, but there are only four patients with LCX as the IRA, and the results must therefore be interpreted cautiously because of large individual variability of coronary artery distribution.

## 5. Clinical Implication

Our automated method provides fast and reliable measurements of myocardium at risk or endocardial extent of infarction, scar size, transmurality, and localization of each scar. These data may have a potential clinical role in the assessment or treatment of ventricular arrhythmias and cardiac failure. Combined with coronary angiography, localization of scars may be used for clinical planning of surgical revascularization and potentially alter the specific placement and number of bypass grafts and used for planning of lead placement in patients with ICD and cardiac resynchronization devices [6].

## 6. Limitation

It is a limitation that the LCX territory is assessed with only 4 patients. Scars adherent to the endocardium were included in the current study in order to ensure that only ischemic scars were assessed. Areas of LGE between endo- and epicardium without a subendocardial origin are therefore excluded. This selection of LGE reduces the risk of the erroneous inclusion of artifacts, but we cannot exclude the possibility that some parts of the LGE areas were due to nonischemic myocardial pathology adherent to endocardium or that some ischemic scars not adherent to endocardium were excluded. The images are hampered by partial volume effects, but the use of relative values compensates to some degree for this drawback.

It is also a limitation that only slices containing myocardium in all 360° have been selected because of the complex mixing of myocardium and connective tissue at the base of the heart, especially the septum [1]. It is also a limitation that all apical segments were visually evaluated to determine if segment 17 had scar.

## 7. Conclusion

This study demonstrates the applicability by use of an automatic method to assess the localization, size, transmurality, and endocardial extent of ischemic scars of LGE-CMR images.

## Figures and Tables

**Table 1:** Baseline characteristics of the study population (*n* = 34) with single-vessel coronary artery disease.

	Total	Infarct related artery (IRA)			*p* value^*∗*^
		LAD (*n* = 15)	RCA (*n* = 15)	LCX (*n* = 4)	
Age (years)	60 ± 13	60 ± 14	59 ± 11	63 ± 17	0.817
Male/female	27/7	13/2	12/3	2/2	0.272
History					
Current smoker	15/28	4/13	8/11	3/4	0.079
Hypertension	9/34	4/15	4/15	1/4	0.997
Diabetes mellitus	1/34	1/15	0/15	0/4	0.497
Preinfarct angina	13/34	5/15	6/15	2/4	0.816
Time to revascular.^*∗∗*^	241 ± 172	312 ± 215	186 ± 101	167 ± 95	0.135
Cholesterol (mmol/L)	5.5 ± 1.1	5.2 ± 0.6	5.9 ± 1.3	5.2 ± 1.0	0.316
HDL-cholesterol (mmol/L)	1.3 ± 0.3	1.4 ± 0.4	1.3 ± 0.3	1.3 ± 0.2	0.903
Lesion location					
Proximal IRA lesion	18	8	7	3	0.601
Mid-IRA lesion	13	7	5	1	0.637
Distal IRA lesion	3	0	3	0	0.124

Treatment					
PCI	34	15	15	4	1.000
*β*-blockers	18	8	8	2	0.992
ACE-inh./AT2 blockers	28	15	12	1	0.161
Aldosterone antagonists	6	6	0	0	0.010

^*∗*^ *p* value calculated by the Kruskal-Wallis test for continuous variables and by the Chi-square test for categorical variables. ^*∗∗*^Time in minutes from start of chest pain to complete revascularization. IRA = infarct related artery. LAD = left anterior descending artery. RCA = right coronary artery. LCX = left circumflex artery. ACE-inh. = angiotensin-converting-enzyme inhibitor. AT2 = angiotensin type 2 receptor. LV = left ventricle. Data are expressed as absolute numbers or mean ± SD.

**Table 2:** Cardiac magnetic resonance characteristics assessed one year following acute myocardial infarction treated by primary percutaneous coronary intervention.

	Total	Infarct related artery (IRA)			*p* ^*∗*^
		LAD (*n* = 15)	RCA (*n* = 15)	LCX (*n* = 4)	
LGE-CMR data					
LVEF (%)	52 ± 11	47 ± 11	56 ± 10	53 ± 5	0.130
LVEDVi (mL/m^2^)	92 ± 23	98 ± 24	91 ± 23	75 ± 11	0.120
LVESVi (mL/m^2^)	46 ± 21	53 ± 24	42 ± 19	35 ± 6	0.110
Scar size as %LV analyzed	7.8 ± 5.5	9.5 ± 6.3	5.8 ± 4.3	8.7 ± 5.2	0.137
Mean scar (pixels)	113 ± 77	118 ± 84	107 ± 78	116 ± 63	0.882
Mean Δ*ϕ*	44 ± 26°	49 ± 31°	37 ± 22°	46 ± 17°	0.571
Endocardial extent as %LV circumference	12.1 ± 7.2%	13.6 ± 8.6%	10.4 ± 6.2%	12.9 ± 4.8%	0.571
Mean Δ*λ*	52 ± 20%	52 ± 0.18%	52 ± 0.24%	56 ± 0.15%	0.959
Ratio scar area/sector with scar	0.42 ± 0.14	0.45 ± 0.15	0.38 ± 0.12	0.46 ± 0.19	0.337
Manual data					
Scar as %LV analyzed	17.4 ± 8.6	19.2 ± 11.2	15.2 ± 6.3	18.4 ± 1.0	0.506
Mean Δ*ϕ*	124 ± 47°	154 ± 53°	101 ±25°	101 ± 11°	0.006^*∗∗*^
Endocardial extent of infarction as %LV circumference	34.6 ± 13.0	42.9 ± 14.7	28.0 ± 6.8	28.0 ± 3.2	0.006^*∗∗*^
Mean Δ*λ*	77 ± 23%	83 ± 17%	78 ± 19%	58 ± 14%	0.065

^*∗*^Kruskal-Wallis. ^*∗∗*^Mann-Whitney test significant for the difference of mean Δ*ϕ* and endocardial extent of infarction as %LV circumference values of patients with LAD and RCA as IRA (*p* = 0.006 and *p* = 0.002, resp.). LAD = left anterior descending artery. RCA = right coronary artery. LCX = left circumflex artery. LVEF = left ventricular (LV) ejection fraction. LVEDVi = LV end-diastolic volume index. LVESVi = LV end-systolic volume index. Δ*ϕ* = endocardium extent of infarction as angle. Δ*λ* = transmurality of the scar as % of LV thickness.

**Table 3:** The table shows the number of times (expressed as percentage) each cardiac segment has a scar. The data is presented according to the infarct related artery (IRA) and segments are evaluated by automatic and manual assessment.

Segment	LAD		RCA		LCX	
	Automatic	Manual	Automatic	Manual	Automatic	Manual
1	99^*∗*^	97^*∗*^	1^†^	0^†^	0^†^	3^†^
2	99^*∗*^	100^*∗*^	1^†^	0^†^	0^†^	0^†^
3	36^§^	39^§^	63^‡^	61^‡^	1^†^	0^†^
4	0^†^	0^†^	93^*∗*^	93^*∗*^	7^†^	7^†^
5	0^†^	0^†^	70^‡^	69^‡^	30^§^	31^§^
6	18^†^	29^§^	18^†^	15^†^	64^‡^	56^∧^
7	100^*∗*^	100^*∗*^	0^†^	0^†^	0^†^	0^†^
8	100^*∗*^	100^*∗*^	0^†^	0^†^	0^†^	0^†^
9	71^‡^	63^‡^	29^§^	37^§^	0^†^	0^†^
10	24^§^	0^†^	67^‡^	85^*∗*^	9^†^	15^†^
11	10^†^	2^†^	41^∧^	60^‡^	49^∧^	38^§^
12	89^*∗*^	71^‡^	0^†^	10^†^	11^†^	19^†^
13	100^*∗*^	100^*∗*^	0^†^	0^†^	0^†^	0^†^
14	96^*∗*^	94^*∗*^	4^†^	6^†^	0^†^	0^†^
15	68^‡^	60^‡^	23^§^	27^§^	9^†^	13^†^
16	61^‡^	69^‡^	32^§^	12^†^	7^†^	19^†^
17	100^*∗*^	100^*∗*^	0^†^	0^†^	0^†^	0^†^

^*∗*^80–100%; ^‡^60–80%; ^∧^40–60%; ^§^20–40%; ^†^0–20%.

LAD = left anterior descending artery. RCA = right coronary artery. LCX = left circumflex artery.
